# Comparison of the Success of Two Techniques for the Endotracheal Intubation with C-MAC Video Laryngoscope Miller Blade in Children: A Prospective Randomized Study

**DOI:** 10.1155/2016/4196813

**Published:** 2016-05-15

**Authors:** Renu Sinha, Ankur Sharma, Bikash Ranjan Ray, Ravinder Kumar Pandey, Vanlalnghka Darlong, Jyotsna Punj, Chandralekha Chandralekha, Ashish Datt Upadhyay

**Affiliations:** ^1^Department of Anaesthesiology, Pain Medicine and Critical Care, All India Institute of Medical Sciences (AIIMS), New Delhi 110029, India; ^2^Department of Biostatistics, All India Institute of Medical Sciences (AIIMS), New Delhi 110029, India

## Abstract

*Background*. Ease of endotracheal intubation with C-MAC video laryngoscope (VLS) with Miller blades 0 and 1 has not been evaluated in children.* Methods*. Sixty children weighing 3–15 kg with normal airway were randomly divided into two groups. Intubation was done with C-MAC VLS Miller blade using either nonstyletted endotracheal tube (ETT) (group WS) or styletted ETT (group S). The time for intubation and total procedure, intubation attempts, failed intubation, blade repositioning or external laryngeal maneuver, and complications were recorded.* Results*. The median (minimum/maximum) time for intubation in group WS and group S was 19.5 (9/48) seconds and 13.0 (18/55) seconds, respectively (*p* = 0.03). The median (minimum/maximum) time for procedure in group WS was 30.5 (18/72) seconds and in group S was 24.5 (14/67) seconds, respectively (*p* = 0.02). Intubation in first attempt was done in 28 children in group WS and in 30 children in group S. Repositioning was required in 14 children in group WS and in 7 children in group S (*p* = 0.06). There were no failure to intubate, desaturation, and bradycardia in both groups.* Conclusion*. Styletted ETT significantly reduces time for intubation and time for procedure in comparison to nonstyletted ETT.

## 1. Introduction

Airway-related problems are among the most common perioperative critical incidents in pediatric anesthesia, and in infants these incidents are four times more common than in older children. In pediatric airway management, both knowledge and training are mandatory [1]. Time for an intubation in children is also limited due to the higher oxygen consumption. Although direct laryngoscope is one of the most commonly used devices for airway management, it requires a direct line of sight to view glottis, which is not always possible in a difficult airway case scenario.

In adults, studies have shown the superiority of intubation conditions with video laryngoscope (VLS) in comparison to direct laryngoscopy in normal as well as difficult airway. It has been observed that VLS provides a better laryngeal view than direct laryngoscopy in children or pediatric mannequins with normal or difficult airways; however, time for intubation is more prolonged along with increased intubation failure [2–5].

The C-MAC VLS system (Karl Storz GmbH & Co. KG, Tuttlingen, Germany) is a new device with different sized blades including Miller 0 and 1 blades [6]. In children, very few studies compared the C-MAC VLS with the conventional Macintosh laryngoscope and other VLSs [7, 8]. Though stylet guides the endotracheal tube (ETT) towards the glottic opening during indirect laryngoscopy, its usefulness with C-MAC VLS Miller blade in children has not been evaluated to date [9]. Therefore, we planned this randomized prospective study with the primary objective to compare time for endotracheal intubation with C-MAC VLS Miller blade using styletted or nonstyletted ETT in the children.

## 2. Material and Methods

This prospective, randomized study was approved by the institutional ethics committee (Ref. number IEC/NP-193/05-06-2014, RP-30/2014) and was registered with the Clinical Trials Registry of India.

### 2.1. Study Population

Sixty children of either sex weighing 3–15 kg scheduled for surgery under general anesthesia were included in the study. The exclusion criteria were children with congenital anomalies, difficult airway, heart disease, reactive airway disease, and metabolic disease and children weighing less than 3 kg or more than 15 kg.

Preanesthetic checkup was done one day prior to the proposed day of surgery and all the children were kept fasting, according to standard fasting guidelines. An informed written consent was obtained from the child's guardian at the time of preanesthetic checkup.

In the operation theatre (OT), C-MAC VLS Miller blade (sizes 0 and 1) availability, charging status, and proper functioning of equipment were ensured. Children were randomized into two groups (group S and group WS) based on computer generated random number table. Sealed opaque envelopes were opened in the OT and intubation technique was decided according to the particular randomized group. In the OT, routine monitors [electrocardiogram, noninvasive blood pressure, and pulse oximetry (SpO_2_)] were attached to the child. Anesthesia was induced with 100% O_2_ and graded increase in sevoflurane concentration with close circuit system. After attaining adequate depth of anesthesia, an intravenous line was secured. Once assisted ventilation was possible, 1 *μ*g kg^−1^ fentanyl along with 0.5 mg kg^−1^ atracurium was administered intravenously. Child was manually ventilated for 3 minutes with monitoring of vital parameters. Meanwhile, appropriate sized endotracheal tube was selected and in group S, a Satin-Slip Intubating Stylet (Mallinckrodt Medical, USA) was inserted in the ETT and normal curvature of ETT was maintained. For group WS, trachea was intubated without stylet. C-MAC VLS Miller blade was inserted through midline and the tongue was depressed along with lifting of epiglottis with the Miller blade tip. After visualization of glottis, endotracheal intubation was attempted with styletted or nonstyletted ETT according to the randomized group (group WS or group S). After successful intubation of trachea, laryngoscope blade was removed and ETT was connected to a close circuit and was ventilated with 100% oxygen. Bilateral equal air entry was checked and ETT was secured.

At any point during procedure, if SpO_2_ decreased less than 94%, manual ventilation with 100% oxygen was resumed. If intubation was unsuccessful even after 2 attempts, it was considered as failure. Again, mask ventilation was resumed to achieve SpO_2_ of 97–100%, if required. Third attempt was done with the other group technique, that is, in group S without stylet and vice versa. If intubation was not possible by both methods, it was considered as unsuccessful and intubation was done with direct laryngoscopy with Miller blade.

In the present study, the primary outcomes were to evaluate time for intubation and time for procedure. Time for intubation was defined as touching the tip of VLS Miller blade to lip till ETT passed through the glottis. Time for procedure was defined as touching the tip of VLS Miller blade to lip till etCO_2_ trace was seen on the monitor. The secondary outcomes recorded were number of attempts at intubation, failed intubation, application of repositioning or external laryngeal maneuver, and complication, that is, desaturation and bradycardia (heart rate less than 20% of baseline). Repositioning was defined as change of position of C-MAC VLS Miller blade. External maneuver such as ETT rotation and application of cricoid manipulation were done as required and recorded.

### 2.2. Statistical Analysis

On the basis of pilot survey of five children in each group, the average time for intubation in group WS was 23.56 ± 14 seconds and in group S was 13.10 ± 10 seconds. On the basis of the results of pilot study, 29 children in each group were required for 5% level of significance and 90% power.

Data was analysed by Stata 11.2 software and presented in median (interquartile range and minimum/maximum). Categorical variable were compared by Pearson Chi-square test/Fisher's exact test. Continuous variables following the normal distribution were compared by independent *t*-test/Wilcoxon rank sum test (skewed data). A *p* value < 0.05 was considered significant.

## 3. Results

**Figure 1:**
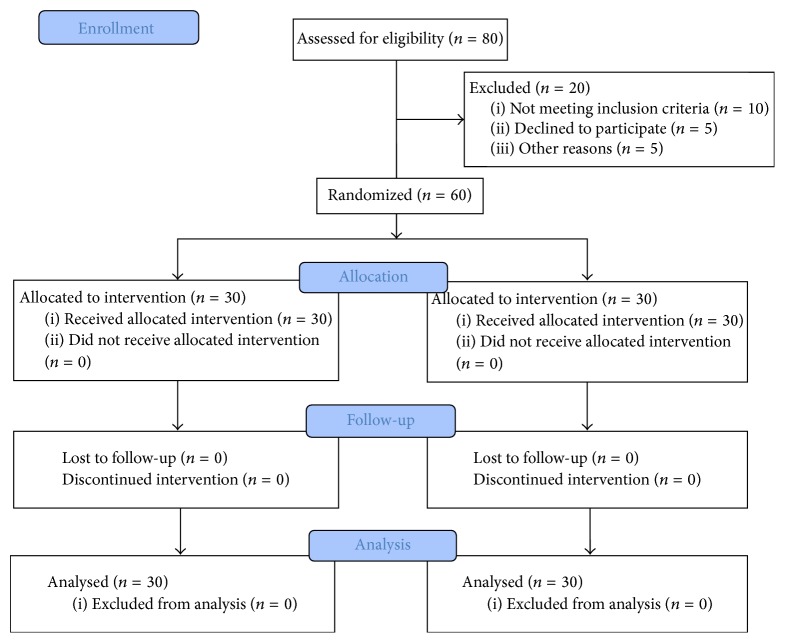
Consort diagram.

Sixty children (30 in each group) undergoing ophthalmic surgeries were enrolled and completed the study (Figure 1). Patient demographic characteristics (age, sex, and weight) for the two groups were comparable (Table 1).

**Figure 2:**
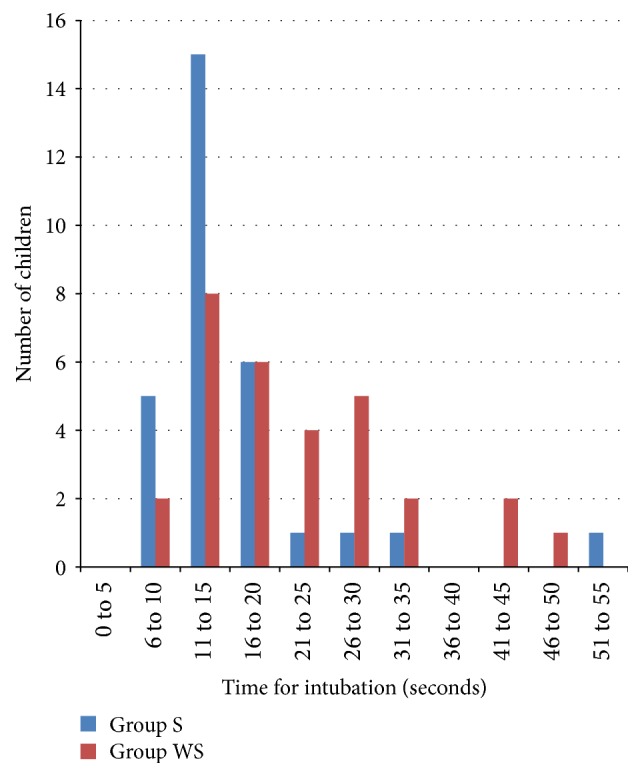
Distribution plot showing time for intubation in group WS and group S.

**Figure 3:**
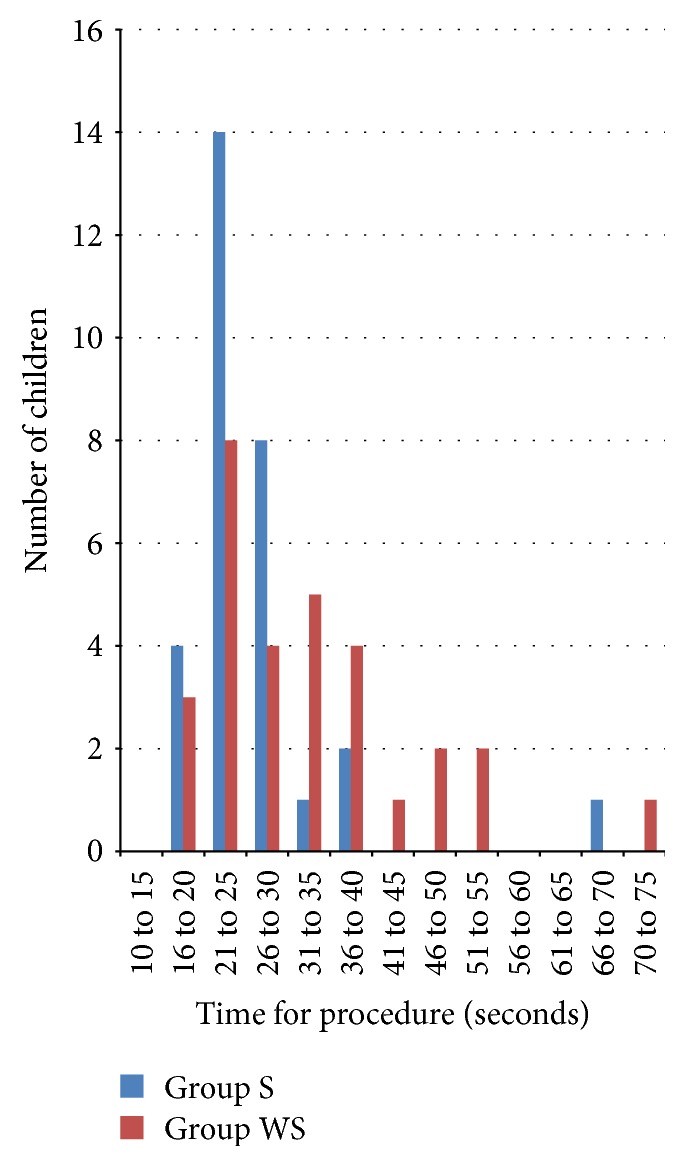
Distribution plot showing time for procedure in group WS and group S.

The median (interquartile range [minimum/maximum]) time for intubation was 19.5 (13.5, 26.0 [9/48]) seconds in group WS and 13.0 (11.0, 19.0 [8/55]) seconds in group S which was statistically significant (*p* = 0.03). The median (interquartile range [minimum/maximum]) time for procedure was 30.5 (22.8, 38.5 [18/72]) seconds in group WS and 24.5 (21.0, 27.3 [14/67]) seconds in group S (*p* = 0.02) which was also statistically significant (Figures 2 and 3).

In group WS, 28 children were intubated in the first attempt and two children required second attempt; in group S, all the children were intubated in the first attempt (*p* = 0.15) (Table 2). Repositioning was required in 14 children (46.7%) in group WS and seven children (23.3%) in group S (*p* = 0.06). An external laryngeal maneuver was required in 4 children (13.3%) in group WS and in 2 children (6.6%) in group S (*p* = 0.39). There was no intubation failures, desaturation, or bradycardia in any child in both groups. Blade size number 1 was used in 26 children in group WS and 23 children in group S while blade size number 0 was used in 4 children in group WS and seven children in group S (*p* = 0.32).

## 4. Discussion

In the present study, the time for endotracheal intubation and total procedure was significantly shorter with the styletted ETT in comparison to that without styletted ETT (*p* < 0.05) with C-MAC VLS Miller blade.

Apart from case reports and series, there are limited studies in literature with C-MAC VLS Miller blade. Storz DCI VLC Miller blade 1 was compared with Miller blade 1 and Macintosh blade 2 in children and it has been observed that though VLS improved glottis visualization in normal airway, time for intubation was prolonged [10]. In contrast, others found that VLS provides better laryngeal view without increasing time for intubation with C-MAC Miller blade 1 in a simulated infant difficult intubation [4]. During comparison of intubation conditions with Miller, Storz Miller, Macintosh, and McGrath Mac curved laryngoscopes in simulated pediatric difficult intubation, straight blades were found to be better than curved blades with additional advantage with Storz Miller blade in terms of glottic view, ease, and duration of intubation and trauma [11].

In different studies, end point of time for intubation has been varied from the appearance of the etCO_2_ trace detection, blade removal from the mouth, first manual ventilation, and so forth [3, 5, 7, 10, 11]. In the present study, time for intubation was taken from the touching of the tip of the VLS Miller blade till ETT passed through the glottis in the monitor, as all intubations were done with VLS. We calculated time from the touching of the tip of blade till etCO_2_ detection as time for procedure.

There are also conflicting case reports and series regarding the advantage of use of styletted ETT in children with normal or difficult airway. Successful intubations in 42 neonates with Storz straight blade video laryngoscope with nonstyletted ETT were reported [12]. Removal of stylet from ETT made intubation possible in a child with Pierre Robin syndrome who had three failed attempts with styletted ETT [13]. In contrast, successful intubation with styletted ETT in children with difficult airway and in a robin sequence using Miller 1 Storz VLS has also been reported when intubation with direct laryngoscope and glidescope was unsuccessful [10, 14]. In our study, intubation was possible in all the children with or without styletted ETT with the advantage of shorter intubation time with styletted ETT. However, longer intubation time in group WS may be attributed to more requirement of repositioning of blade to facilitate intubation in comparison to group S (14 versus 7 children). In addition, stylet makes ETT tougher, which may ease manipulation of smaller ETT in younger infants. However, blind advancement of styletted ETT may increase the chances of airway injury due to protrusion of the stylet tip beyond the end of the ETT. There may be increased risk of inadvertent removal of the ETT during removal of the stylet.

To date, there is no consensus for shape of styletted ETT to be used during intubation with Miller blade Storz VLS. Twelve-degree bend of styletted ETT like the deflection of the tip of the Miller blade Storz VLS has been advised [14]. However, studies either have used 60–70-degree angulation or did not give any details of angulation of styletted ETT [10, 11]. In the present study, styletted ETT was not angulated and kept in normal curvature as it is a routine practice with Miller blade in our institute. In the previous studies, angulation of ETT may be one of the factors for unsuccessful intubation with styletted ETT with C-Mac Miller blade. Further tip of the angulated ETT may hit the anterior wall of trachea during advancement along with tracheal trauma [14].

In the present study, we followed four steps for successful intubation with VLS advised by Holm-Knudsen and did not encounter failed intubation or complications [13]. Hand eye coordination is one of the factors for successful intubation with VLS. In the present study, first author who had good experience with C-MAC VLS intubated all the children to prevent confounding factor.

Method of ETT insertion is also important for a successful intubation. As, during direct laryngoscopy with Miller blade, insertion of ETT through C-shaped groove obstructs the direct vision, that is, 15 degrees from naked eyes, ETT is inserted through the right angle of the mouth and then moves towards the glottis in midline [15]. In contrast, insertion of ETT can be done through the Storz VLS Miller blade groove as it does not obstruct the laryngeal view due to presence of the camera at the light source with 80-degree magnified view [11]. Due to magnified glottic view, even small movement of ETT will be seen as major movement on the monitor. Similar to previous study, we inserted ETT through the Miller blade groove and had 100% successful intubation [12].

Different methods have been used for insertion of Miller blade for direct laryngoscopy ranging from midline to paraglossal approach to improve laryngeal view [15, 16]. We inserted Miller blade through midline compressing the tongue.

Successful intubation also depends on the placement of the tip of the C-MAC VLS Miller blade. Different authors have used different techniques, that is, tip of the blade in the vallecula or lifting the epiglottis with storz VLS Miller blade with variable success rate [11, 13]. As classically practiced with Miller blade during direct laryngoscopy, we lifted the epiglottis with the Miller blade in all the cases to prevent the confounding factor. In our study, in 2 cases of group WS and 3 cases in group S, more time was taken in lifting the epiglottis. In these 5 cases, epiglottis was small and was slipping during lifting.

The limitation of the present study was exclusion of difficult airway.

## 5. Conclusion

To conclude, styletted ETT significantly reduces time for intubation and time for procedure in comparison to nonstyletted ETT in children with C-MAC VLS Miller blade. Both the techniques are safe.

## Figures and Tables

**Table 1:** Patient characteristics. Data are median (interquartile range [minimum/maximum]).

	Group WS (*n* = 30)	Group S (*n* = 30)	*p* value
Age (months)	24 (12, 36 [3/72])	24 (9.8, 36 [3/72])	0.99
Sex (male : female)	22 : 8	21 : 9	0.77

**Table 2:** Comparison of intubation conditions between two groups. Data are median (interquartile range [minimum/maximum]) or number (%) ^*∗*^significant with *p* < 0.05.

	Group WS (*n* = 30)	Group S (*n* = 30)	*p* value
Time for intubation (seconds)	19.5 (13.5–26.0 [9/48])	13.0 (11.0–19.0 [8/55])	0.03^*∗*^
Time for procedure (seconds)	30.5 (22.8–38.5 [18/72])	24.5 (21.0–27.3 [14/67])	0.02^*∗*^
Intubation attempts			
1	28 (93.3%)	30 (100%)	0.15
2	2 (6.7%)	0	
Blade size			
0	4	7	0.32
1	26	23	
Repositioning	14 (46.7%)	7 (23.3%)	0.06
Maneuvers	4 (13.3%)	2 (6.6%)	0.39
Desaturation	0	0	
Bradycardia	0	0	

## Additional Points

What Is Already Known about the Topic. What is already known about the topic is that video laryngoscope helps in intubation and is useful in case of difficult intubation.

What New Information This Study Adds. What new information this study adds is that, to date, there are conflicting reports that use of styletted endotracheal tube with C-MAC video laryngoscope will make intubation easier or difficult. This study concluded that use of styletted endotracheal tube helped in faster intubation in children provided that the curvature of endotracheal tube is not changed.
